# The Use of Apparent Diffusion Coefficient Values for Differentiating Bevacizumab-Related Cytotoxicity from Tumor Recurrence and Radiation Necrosis in Glioblastoma

**DOI:** 10.3390/cancers16132440

**Published:** 2024-07-02

**Authors:** Kamand Khalaj, Michael A. Jacobs, Jay-Jiguang Zhu, Yoshua Esquenazi, Sigmund Hsu, Nitin Tandon, Alireza Akhbardeh, Xu Zhang, Roy Riascos, Arash Kamali

**Affiliations:** 1 Department of Diagnostic and Interventional Imaging, UTHealth Houston, Houston, TX 77030, USA; kamand.khalaj@uth.tmc.edu (K.K.);; 2 The Department of Radiology and Oncology, The Johns Hopkins School of Medicine, Baltimore, MD 21205, USA; 3 Department of Computer Science, Rice University, Houston, TX 77005, USA; 4 The Vivian L. Smith Department of Neurosurgery, McGovern Medical School, UTHealth Houston, Houston, TX 77030, USA; 5 Division of Clinical and Translational Sciences, Department of Internal Medicine, UTHealth, Houston, TX 77030, USA

**Keywords:** MRI, GBM, radiation necrosis, bevacizumab, cytotoxicity, coagulative necrosis, ADC maps

## Abstract

**Simple Summary:**

This study investigates an imaging method for distinguishing different lesions in brain cancer patients using MRI scans. Glioblastoma is a highly aggressive brain tumor, and it is crucial to differentiate between actual tumor recurrence, radiation-induced damage, and side effects from a common treatment called Bevacizumab. Traditional MRI techniques often struggle to make these distinctions, leading to challenges in treatment decisions. This study explores the use of a specific MRI measurement, called Apparent Diffusion Coefficient (ADC) values, to improve diagnostic accuracy. By identifying unique patterns in ADC values, this method could help better determine the nature of brain lesions, ultimately leading to more precise and effective treatment plans for glioblastoma patients.

**Abstract:**

Objectives: Glioblastomas (GBM) are the most common primary invasive neoplasms of the brain. Distinguishing between lesion recurrence and different types of treatment related changes in patients with GBM remains challenging using conventional MRI imaging techniques. Therefore, accurate and precise differentiation between true progression or pseudoresponse is crucial in deciding on the appropriate course of treatment. This retrospective study investigated the potential of apparent diffusion coefficient (ADC) map values derived from diffusion-weighted imaging (DWI) as a noninvasive method to increase diagnostic accuracy in treatment response. Methods: A cohort of 21 glioblastoma patients (mean age: 59.2 ± 11.8, 12 Male, 9 Female) that underwent treatment with bevacizumab were selected. The ADC values were calculated from the DWI images obtained from a standardized brain protocol across 1.5-T and 3-T MRI scanners. Ratios were calculated for rADC values. Lesions were classified as bevacizumab-induced cytotoxicity based on characteristic imaging features (well-defined regions of restricted diffusion with persistent diffusion restriction over the course of weeks without tissue volume loss and absence of contrast enhancement). The rADC value was compared to these values in radiation necrosis and recurrent lesions, which were concluded in our prior study. The nonparametric Wilcoxon signed rank test with *p* < 0.05 was used for significance. Results: The mean ± SD age of the selected patients was 59.2 ± 11.8. ADC values and corresponding mean rADC values for bevacizumab-induced cytotoxicity were 248.1 ± 67.2 and 0.39 ± 0.10, respectively. These results were compared to the ADC values and corresponding mean rADC values of tumor progression and radiation necrosis. Significant differences between rADC values were observed in all three groups (*p* < 0.001). Bevacizumab-induced cytotoxicity had statistically significant lower ADC values compared to both tumor recurrence and radiation necrosis. Conclusion: The study demonstrates the potential of ADC values as noninvasive imaging biomarkers for differentiating recurrent glioblastoma from radiation necrosis and bevacizumab-induced cytotoxicity.

## 1. Introduction

Glioblastomas are the most common aggressive primary brain neoplasm, constituting approximately 49% of all malignant brain tumors in adults [1,2]. The incidence of glioblastoma increases after the age of 40 and peaks in adults aged 75 to 84 years, and it has a median survival of less than two years [3]. Magnetic Resonance Imaging (MRI) plays a pivotal role in the initial assessment of suspected brain malignancies using advanced sequences of T2-imaging, Diffusion-Weighted Imaging, Apparent Diffusion Coefficient (ADC) mapping (DWI/ADC) and T1 contrast-enhanced Imaging [4]. Most centers use a standardized brain imaging protocol for accurate evaluation [4,5]. Maximal safe surgical resection prioritizing the preservation of neurologic function is paramount for optimal outcomes. Gross total resection, synonymous with surgical removal of most if not all contrast-enhancing tumors has been associated with improved progression-free survival and overall survival rates [6]. Additionally, recent studies have shown that supratotal resection is associated with improved outcomes [7]. Postoperatively, chemoradiation, a combination of radiation therapy and the oral alkylating agent temozolomide, is typically administered to patients [8].

Glioblastoma recurrence is a major challenge, with a median progression-free survival of approximately seven months [8]. In this landscape, bevacizumab, a monoclonal antibody targeting human vascular endothelial growth factor, has gained importance for its role in controlling vasogenic edema, a common symptom in glioblastoma patients [9,10,11,12] While bevacizumab has demonstrated efficacy in improving progression-free survival, its impact on overall survival remains a topic of debate [13,14]. 

Emerging multiparametric MR imaging sequences offer promise in detecting infiltrating lesions and assessing treatment responses. Diffusion-weighted imaging (DWI) and the derived Apparent Diffusion Coefficient (ADC) maps provide insight into the free diffusion of water molecules in tissues. The ADC values illustrate the degree of diffusion restriction of water molecules, presenting quantitative values in units of mm^2^/s. These features render ADC value as a potential tool for characterizing lesion behavior [15]. ADC values have also been used for monitoring treatment response and survival prediction [14,16,17,18,19]. 

A recent study by our group demonstrated the potential of ADC values to distinguish between radiation necrosis and tumor progression by taking a new approach. Unlike prior studies that measured the ADC values of the enhancing component, this study assessed the ADC values of the areas of restricted diffusion irrespective of tissue enhancement. Notably, the results showed a range of ADC values, with post-treatment ischemic changes exhibiting the lowest values (250.1 ± 57.2 mm^2^/s), progressive tumor displaying highest values (752.8 ± 132.5 mm^2^/s), and post-treatment radiation necrosis having a middle range (479.0 ± 105.2 mm^2^/s). Importantly, statistical analyses demonstrated significant differences between ADC values in these conditions [20]. A subset of glioblastoma patients treated with bevacizumab may develop areas of diffusion restrictions, which are called bevacizumab-induced cytotoxicity [14].

Distinguishing between the glioblastoma recurrence, radiation necrosis, and bevacizumab-induced cytotoxicity continues to present a diagnostic challenge for oncologists, radiologists, and neurosurgeons given the presence of diffusion restrictions in all three groups in conventional MRI exams. Research indicates that patients who exhibit stable diffusion-restricted lesions after undergoing bevacizumab therapy experience increased survival while progressive diffusion restriction is associated with decreased survival [14,21]. In the presence of bevacizumab, the immature and weak vasculature of the tumor is stabilized, and the rate of microvascular proliferation and BBB (blood–brain barrier) permeability decreases. These microstructural and functional changes translate into a dramatic and almost immediate reduction in tumor enhancement on MRI scans [22]. Conflicting studies have identified the areas of restricted diffusion to represent areas of necrosis, hypercellular lesion or bevacizumab-induced cytotoxicity, highlighting the need for precise differentiation [14,21,23,24,25,26]. Glioblastomas are primarily characterized by liquefactive necrosis, leading to higher ADC values secondary to higher amounts of diffusion and corresponding hyperintensity on ADC images [27]. In contrast, radiation necrosis generally consists of coagulative necrosis which presents as firm tissue with ghostly cellular remnants, resulting in lower ADC values [27]. This clinical backdrop underscores the critical need for a noninvasive tool to differentiate between bevacizumab-related cytotoxicity, radiation necrosis, and recurrent glioblastoma. Clinically, accurately distinguishing between these three entities is important and directly influences patient management and treatment strategies. Tumor progression typically necessitates either re-resection, Gamma knife or additional radiation therapy. Whereas radiation necrosis is often managed with bevacizumab or steroids to control edema and prevent herniation of the brain [2,8]. Conversely, bevacizumab-related cytotoxicity generally does not require intervention unless it causes a mass effect or herniation, which would necessitate steroid therapy [2,8,14]. Misidentification of these conditions could lead to inappropriate treatment, unnecessary invasive procedures, or delayed appropriate care, significantly impacting patient outcomes and quality of life. The aim of this study is to investigates the sensitivity and specificity of ADC values as a noninvasive imaging biomarker to differentiate treatment-related changes from tumor progression. 

## 2. Materials and Methods

### 2.1. Study Population

This retrospective study, approved by the local Institutional Review Board, involved 21 patients diagnosed with histologically confirmed recurrent IDH-wild type glioblastoma (WHO 2021) who underwent bevacizumab therapy and standard of care (SOC) surgery and chemoradiation. Follow-up MRIs were obtained every eight weeks postradiation therapy or earlier if patients were symptomatic. 

**Figure 1:**
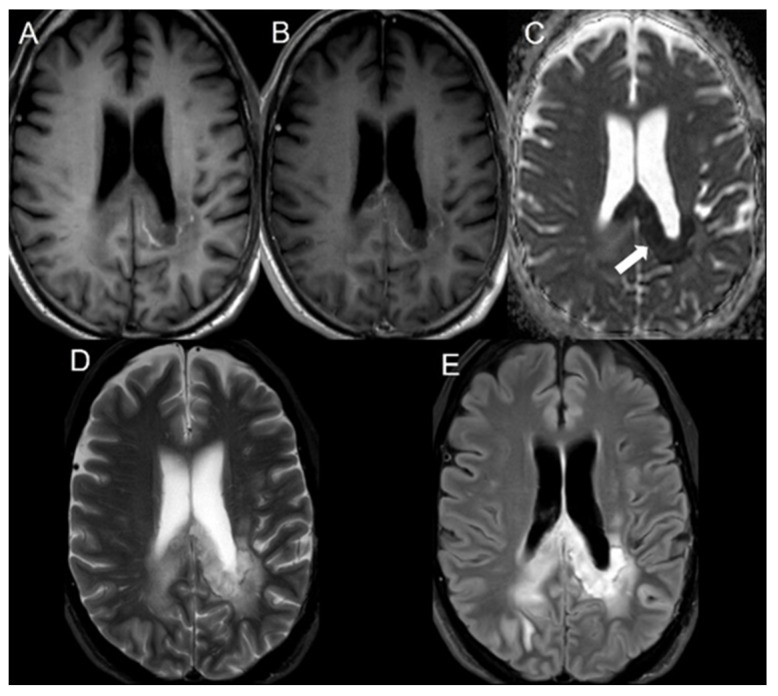
A 59 y/o male with bevacizumab-induced cytotoxicity. Axial MRI of the brain including (**A**) T1 weighted image, (**B**) postcontrast T1 weighted image, (**C**) ADC map, (**D**) T2 weighted image, and (**E**) FLAIR. The lesion demonstrates no contrast enhancement in the splenium of the corpus callosum (**B**) with restricted diffusion on the ADC map (white arrow in (**C**)). Mild adjacent areas of edema are seen in T2 weighted and FLAIR sequences (**D**,**E**).

Diffusion restriction features were defined as areas of lesion showing increased signal intensity on DWI and corresponding hypointense signal on the ADC maps. The bevacizumab-induced cytotoxicity was diagnosed based on characteristic imaging features in brain MRI. The imaging diagnosis involved well-defined regions of restricted diffusion with persistent diffusion restriction over the course of weeks without tissue volume loss (to rule out ischemic infarcts) and an absence of contrast enhancement (to rule out radiation necrosis) (Figure 1) [21]. The mean ADC and rADC values of bevacizumab-induced cytotoxicity lesions were compared to the mean ADC and rADC values of radiation necrosis and tumor progression, the values of which were documented in our previous study [20].

### 2.2. MRI Parameters and Postprocessing

**Figure 2:**
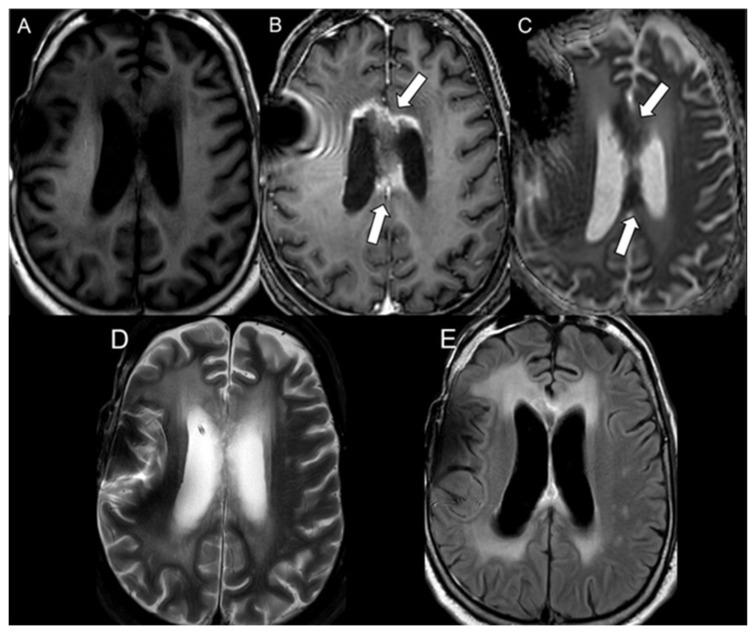
A 61 y/o male with radiation necrosis. Axial MRI of the brain including (**A**) T1 weighted image, (**B**) T1 weighted postcontrast image, (**C**) ADC map, (**D**) T2 weighted image, and (**E**) FLAIR. The lesion demonstrates some contrast enhancement within the body of the corpus collosum and fornix (white arrows in (**B**)) and restricted diffusion on the ADC map (white arrows in (**C**)).

**Figure 3:**
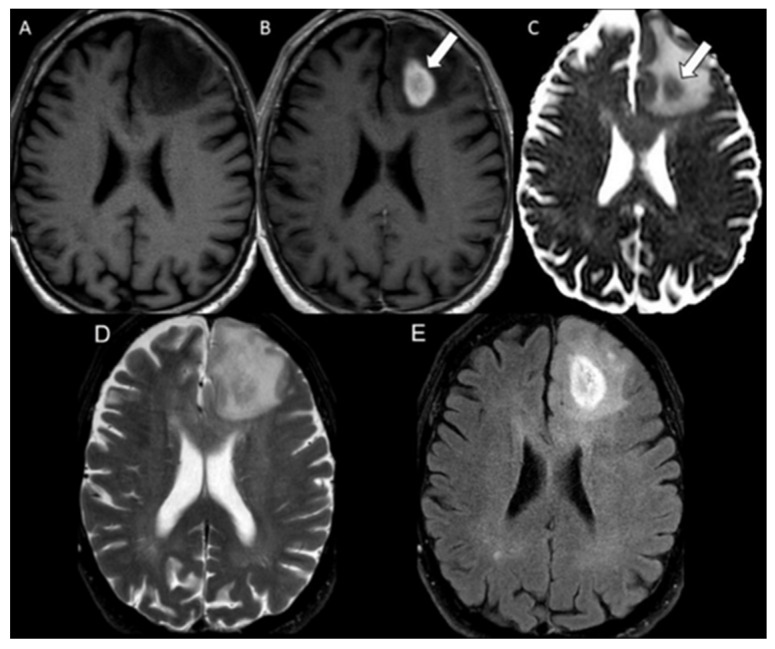
An 86 y/o male with tumor progression. Axial MRI of the brain including (**A**) T1 weighted image, (**B**) postcontrast T1 weighted image, (**C**) ADC map, (**D**) T2 weighted image, and (**E**) FLAIR. The lesion demonstrates central contrast enhancement in the left frontal lobe (white arrow in (**B**)) with corresponding restricted diffusion on the ADC map (white arrow in (**C**)) and areas of surrounding T2/FLAIR hyperintensity (**D**,**E**).

**Figure 4:**
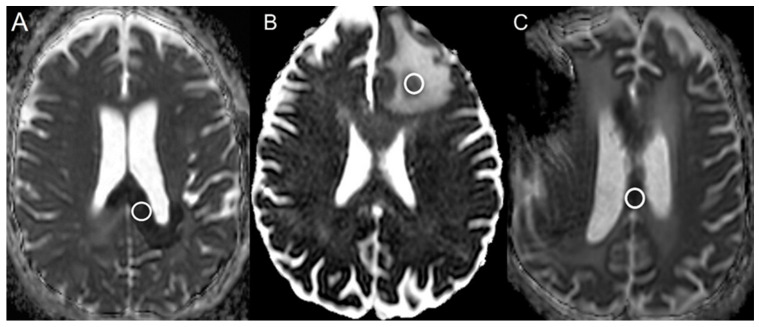
Apparent Diffusion Coefficient maps from different patients with (**A**) bevacizumab-induced cytotoxicity, (**B**) lesion (glioblastoma) progression, and (**C**) radiation necrosis. The white circles were the regions selected from the largest ADC hypointensity for evaluation of the ADC values.

Imaging data were acquired using either a 1.5-T or a 3T MRI GE scanner. The B-values (b = 0 and 1000 s/mm^2^) were acquired along the three orthogonal diffusion gradients (x,y,z) with the imaging protocol as follows: field strength = 1.5 and 3.0 T, TE = 60–90 ms, TR = 3500–7200 ms, matrix = 160 × 110 to 240 × 240, Fov = 24 × 24 cm, slice thickness/slice gap = 3–5 mm/0–1 mm, scan time = 1 to 3.6 min, and parallel imaging factor = 2. Automated generation of ADC maps were performed at the scanners (Figure 1, Figure 2 and Figure 3). ADC values were then extracted by two blinded readers: a board-certified neuroradiologist with a decade of experience and a trained research associate physician. In cases of discrepancy between the two readers, a consensus was reached based on the senior reader’s opinion. Regions of interest (ROIs) were placed in the areas of greatest hypointensity within the lesion using the ADC map. Cystic, hemorrhagic, or necrotic regions were excluded using other imaging sequences (Figure 4). Lesions greater than 1 cm were selected for ADC measurement to minimize variability in ROI selection. A reference ROI was delineated within the normal-appearing white matter on the contralateral hemisphere for standardization of ADC values. Ratios of lesion ADC to normal appearing contralateral white matter tissue ADC (L/WM) were calculated from the equation on the lesion and white matter tissue.

$$Normalized\;ADC\;value=\frac{ADC\;value\;of\;Lesion}{ADC\;value\;of\;Contralateral\;White\;Matter}$$

All ADC values are presented in units of 1 × 10^−6^ mm^2^/s.

### 2.3. Statistical Analysis

Statistical analysis was conducted using SAS 9.4 (SAS Institute Inc., Cary, NC, USA). Apparent diffusion coefficient (ADC) values, treated as continuous variables, were expressed as means and standard deviations (SDs). We performed the Shapiro–Wilk test and depicted histograms to examine normality. These two variables exhibited slightly skewed distributions in the glioblastoma group. We utilized nonparametric tests in all evaluations to obtain conservative statistical inferences. Comparison between the 3 groups was addressed via the Wilcoxon rank sum test. Comparison between lesions and normal white matter was evaluated via Wilcoxon signed rank test. A *p*-value ≤ 0.05 was considered statistically significant. In the Supplementary Materials, the descriptive statistics of ADC map values, including the median, first and third quartiles, skewness, and kurtosis are shown. We implemented the bootstrap method to find the percentile confidence interval for an AUC, as well as estimate the *p*-value for the difference in skewness (or kurtosis) statistics between the lesion and normal-appearing white matter (Table S1).

## 3. Results

**Figure 5:**
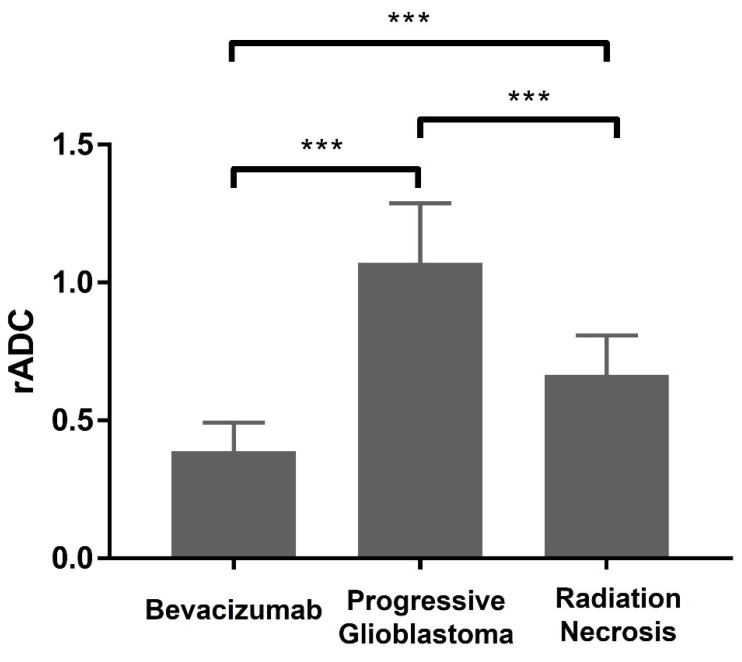
Mean values of rADC in the bevacizumab-induced cytotoxicity, progressive glioblastoma, and radiation necrosis lesions [*** *p* < 0.001. Data are presented as mean ± SD].

Table 1 displays patient characteristics and clinical parameters for those receiving bevacizumab. The mean ADC value for bevacizumab-induced cytotoxicity was 248.1 ± 67.2, whereas normal white matter had a mean ADC value of 647.2 ± 94.6. Lesion progression had a mean ADC value of 752.8 ± 132.5, while normal-appearing contralateral white matter was 709.2 ± 63.5. For radiation changes, the mean ADC value was 479.0 ± 105.2, compared to a reference value of 723.3 ± 64.0. ADC values yielded perfect or nearly perfect differentiation for bevacizumab-induced cytotoxicity and radiation necrosis from normal-appearing white matter, with an AUC of 1.0 and 0.98, respectively. For patients with recurrent glioblastoma, the ADC values were not differentiable between the lesion and normal-appearing white matter (AUC = 0.59, 95% CI 0.41–0.70). The mean ADC values and the AUC estimates are summarized in Table 2 and Table 3. The rADC values are shown in Table 4. The rADC values differed significantly among the three groups (*p* value < 0.001; for all comparisons, see Figure 5). Figure 5 illustrates the comparison between mean rADC values, which were significantly higher in tumor progression (1.07 ± 0.22) compared to radiation necrosis (0.66 ± 0.14) and bevacizumab-induced cytotoxicity (0.39 ± 0.10). Additionally, radiation-induced changes had significantly higher rADC values compared to areas of bevacizumab-induced cytotoxicity. The mean time intervals between the initiation of bevacizumab therapy and the appearance of diffusion restriction, the initiation of therapy and death, and the appearance of diffusion restriction and death were 119.8 days (range, 16–371), 409 days (range, 75–878 days), and 299 days (range, 16–829 days), respectively. In these patients who underwent bevacizumab therapy, diffusion restriction was identified in the corpus callosum (12 patients), periventricular white matter (7 patients), and corona radiata (3 patients) (Table 1). Finally, The Normality for ADC and relative ADC variables was satisfactory in both the bevacizumab-induced coagulative necrosis and radiation necrosis groups.

## 4. Discussion

We have demonstrated that the ADC values of lesions characterized by bevacizumab-induced restricted diffusion were significantly lower than the ADC values of radiation necrosis or GBM recurrence. Moreover, using the ADC ratio clearly shows the differentiation between the different groups of treatment responses. 

In 20–60% of patients treated with bevacizumab, a phenomenon characterized by a pseudoresponse is observed, credited to its stabilizing impact on the BBB [28]. Pseudoresponse is defined by decreased enhancing tissues following bevacizumab therapy but constrained overall survival rates. In cases where the diagnosis is clinically uncertain, invasive procedures such as biopsies are often necessary.

Post-treatment ischemic changes usually occur due to acute vascular cut off during operation and can result in sudden tissue death secondary to acute hypoxia and lead to eventual tissue shrinkage and volume loss in few weeks. However, the bevacizumab-induced cytotoxicity demonstrates areas of coagulative necrosis and chronic hypoxia [25,29] with no apparent volume loss over the course of weeks to months [25].

A recent meta-analysis review showed that at the group level, the ADC values can effectively differentiate between tumor progression and treatment-related abnormalities in post-treatment glioma patients (*p* = 0.005), with combined sensitivities and specificities reaching 85% and 81%. However, this study reported that treatment-related changes had a higher ADC value compared to tumor progression in the literature, which was different than our findings [30]. This can be due to the differences in the ROI selection criteria of the studies included in the meta-analysis compared to the current study. Those studies only included the areas of enhancement showing diffusion restriction as opposed to the current study which included enhancing and nonenhancing lesions demonstrating diffusion restrictions. Multiple recent studies have also investigated lesions demonstrating diffusion restriction in glioblastoma patients. Gupta et al. observed that diffusion restriction preceded the emergence of an enhancing lesion in a subgroup of glioblastoma patients, regardless of bevacizumab therapy; this suggested that such diffusion restriction indicated lesion hypercellularity [24]. Gerstner et al. showed nonenhancing tumor existence at the location of diffusion restrictions in glioblastomas confirming the importance of inclusion of ADC values of nonenhancing-lesions in treated glioblastoma patients [31]. Pope et al., in their investigation using histogram analysis of low ADC values, concurred with the notion that these values signified areas of heightened cellularity [32]. On the contrary, some studies proposed a different hypothesis, suggesting that these regions of diffusion restrictions might not be indicative of tumor hypercellularity, since not all patients’ conditions progressed [25,33]. Instead, they theorized that the areas of diffusion restrictions were more likely related to chronic hypoxia and necrosis [33]. In a study conducted by Nguyen et al., postmortem biopsy observations indicated that areas exhibiting diffusion restrictions corresponded to areas of coagulative necrosis [14]. 

Diffusion restriction is recognized as a possible indicator to differentiate treatment response from tumor progression. Previous studies have identified a reverse relationship between ADC values and other parameters such as cellularity, tumor grade, and Ki-67 proliferation indices [24,32]. However, our recent study demonstrated that the ADC values in radiation necrosis were significantly lower than the ADC values in GBM progression [20]. The current study found that bevacizumab-induced coagulative necrosis, which is rare in the brain, is associated with extremely low diffusion values, with a mean value of 0.24 × 10^−3^ mm^2^/s and rADC values of 0.39 ± 0.10. Other studies with very smaller sample sizes compared to the current study suggested ADC values as high as 0.59, 0.63 [34], 0.63 [25], and 0.663 × 10^−3^ mm^2^/s [14] in patients who underwent bevacizumab therapy and no ADC ratios between tissue types were discussed. This difference may be explained by the difference in ROI selections. In the study by Nguyen et al. [14], and Laviolette et al. [34], the ROIs were selected by correlating the diffusion restriction seen on MRI with the necrotic lesion in the pathologic sections of the whole brain. In the study conducted by Rieger et al., the regions of interest were placed on the DWI hyperintensity on contrast-enhancing tumor. In the current study, we selected the ROIs from areas of highly restricted diffusion based on the “darkest” area of the lesion on the ADC map, irrespective of enhancement or nonenhancement within tissues demonstrating diffusion restrictions. 

The mechanism underlying diffusion-restricted necrosis has been theorized in the previous literature. For example, it was suggested that areas of coagulative necrosis might develop due to prolonged hypoxia induced by bevacizumab [25]. These zones were identifiable just four weeks after starting bevacizumab treatment and endured for as long as 80 weeks [25]. Moreover, they were mainly identified along the white matter tracts, particularly in the corpus callosum and corona radiata [24]. Qualitative assessments of patients with bevacizumab-induced cytotoxicity demonstrated that the diffusion restriction was commonly observed around the ventricles and corpus callosum [21,35]. This further emphasizes the importance of ADC values in the differentiation of bevacizumab-related cytotoxicity from radiation necrosis or tumor progression in glioblastoma patients. 

The major finding of this study lies in its specific focus on the cytotoxic effects of bevacizumab within brain MRI scans of treated glioblastoma patients who underwent bevacizumab therapy and developed bevacizumab-related cytotoxicity. Unlike previous studies that broadly examined the effects of bevacizumab, such as its impact on the blood–brain barrier and tumor progression in gliomas [36], this study specifically addressed the differentiation of bevacizumab-related cytotoxicity from radiation necrosis and tumor progression in treated glioblastoma patients. This methodological innovation enhances diagnostic accuracy and potentially reduces the need for invasive procedures such as biopsies. The imaging diagnostic accuracy is critical as the management and treatment of these conditions differ significantly.

Previous studies have highlighted the paradoxical effects of bevacizumab and its role in treatment protocols [37,38]. Our research may bring new insights on how ADC values may be used noninvasively to differentiate post-treatment changes in treated glioblastoma patients, in particular for distinguishing bevacizumab-related cytotoxicity, which typically does not require intervention unless mass effect or herniation of the brain occurs.

Recent preclinical studies have shown that nanoparticle conjugation may enhance the delivery and effectiveness of bevacizumab by improving its ability to cross the BBB and reducing off-target effects in small animals [39,40]. These innovative approaches could potentially improve treatment outcomes. The role of cell-penetrating peptides or other BBB permeability modulators in enhancing bevacizumab treatment efficacy may potentially offer new avenues for improving drug delivery and therapeutic outcomes, as shown in the preclinical studies.

As discussed by van den Elshout et al., there are inherent limitations when utilizing ADC values [30]. ADC values are susceptible to influence from various factors such as scanner type and protocol. However, in this study we standardized the ADC by utilizing the ratio of ADC values (rADC), using the contralateral normal-appearing white matter as a baseline reference. This approach facilitated data comparison across different scanners.

One limitation of the current study was the small number of subjects. However, the current study had the largest sample size of patients with bevacizumab-related cytotoxicity in the literature to date. The diagnosis of bevacizumab-induced coagulative necrosis was based on imaging characteristics of persistent diffusion restrictions lasting longer than six months without tissue volume loss or contrast enhancement. However, the potential of this selection impacting the results was minimal, as no other treatment-related changes exhibited these imaging and tissue characteristics to our knowledge.

Additionally, DNA methylation profiles were unavailable for the majority of glioblastoma patients before 2018, preventing an investigation into the potential effects of DNA methylation on ADC values in glioblastoma patients based on the current results. It is crucial to note that the current study excluded the cystic variant of glioblastoma, a rare entity, and therefore, the results of this study should not be extrapolated to cystic glioblastomas. The current study also did not assess ADC values in glioblastoma patients with mixed tumor recurrence and radiation necrosis, leaving the results for this group unknown at this time. Perfusion sequences and related parameters, which may be a part of standard brain imaging protocol, could potentially provide a better understanding of the underlying pathology of the lesion after treatment. Unfortunately, we had a limited number of subjects with bevacizumab cytotoxicity included in our study, who did not undergo perfusion imaging at our institution. Future studies may include perfusion imaging to investigate the differentiation between bevacizumab-related cytotoxicity, radiation necrosis, and tumor progression. 

## 5. Conclusions

This study demonstrated that ADC values may be a potential noninvasive imaging biomarker capable of discerning between tumor progression and treatment-related alterations related to bevacizumab-induced cytotoxicity and radiation necrosis in glioblastoma patients. Such differentiation could potentially impact clinical decision-making by reducing the necessity for invasive procedures that may carry additional risks to patients. To build on these preliminary findings, larger prospective multicenter studies will be conducted to confirm the use of ADC in conjunction with more advanced imaging techniques, such as perfusion MRI in bevacizumab-induced cytotoxicity. These will extend potential biomarkers and give a more complete picture of treatment-related changes. Additionally, investigating how other newer treatments may affect ADC values could further improve how we monitor and treat these patients. Finally, the addition of radiomics may give more information on both local and global effects of treatments in these types of lesions and treatments. 

## Figures and Tables

**Table 1:** Patient characteristics and clinical parameters for bevacizumab-receiving patients. (CC: Corpus Callosum, CR: Corona Radiata, PV: Periventricular white matter).

Patient No.	Age at Death	Tumor Type	Surgery	XRT	TMZ	Location of Focal Region of Diffusion Restriction	Bevacizumab (Day) before Death	Bevacizumab (Day) before Focal Region Appears	Days between Focal Region and Death
**1**	Still alive	GBM	+	+	+	CC	N/A	181	N/A
**2**	32	GBM	+	+	+	CC	90	74	16
**3**	45	GBM	+	+	+	CC	574	61	513
**4**	77	GBM	+	+	+	CR	424	58	366
**5**	57	GBM	+	+	+	CC	647	339	308
**6**	Still alive	GBM	+	+	+	CC	N/A	258	N/A
**7**	48	GBM	+	+	+	PV	283	16	267
**8**	68	GBM	+	+	+	CR	92	34	58
**9**	67	GBM	+	+	+	CR	573	150	423
**10**	64	GBM	+	+	+	PV	589	25	564
**11**	57	GBM	+	+	+	CC	281	57	224
**12**	67	GBM	+	+	+	CC	75	41	34
**13**	65	GBM	+	+	+	PV	272	90	182
**14**	48	GBM	+	+	+	PV	717	371	346
**15**	51	GBM	+	+	+	CC	229	41	188
**16**	72	GBM	+	+	+	PV	878	49	829
**17**	52	GBM	+	+	+	CC	575	140	435
**18**	54	GBM	+	+	+	CC	651	355	296
**19**	83	GBM	+	+	+	PV	168	36	132
**20**	57	GBM	+	+	+	PV and CC	359	41	318
**21**	47	GBM	+	+	+	Parasagittal frontal lobe, cingulate gyrus, CC	295	99	196

**Table 2:** Descriptive statistics of ADC map values.

Group	N	Lesion	Normal	*p* Value
		Mean ± SD	Mean ± SD	
Bevacizumab	21	248.1 ± 67.2	647.2 ± 94.6	<0.001
Progressive glioblastoma	49	752.8 ± 132.5	709.2 ± 63.5	0.08
Radiation necrosis	58	479.0 ± 105.2	723.3 ± 64.0	<0.001

ADC: Apparent Diffusion Coefficient, SD: Standard Deviation.

**Table 3:** AUC estimates for using ADC values to differentiate lesions from normal-appearing white matter.

Group	AUC	95% CI
Bevacizumab	1.00	N/A
Progressive glioblastoma	0.59	0.41–0.70
Radiation necrosis	0.98	0.95–1.00

AUC: Area Under the ROC Curve.

**Table 4:** Descriptive statistics of rADC values (*p* < 0.001 for all pairwise comparisons).

Group	N	Mean ± SD
Bevacizumab	21	0.39 ± 0.10
Progressive glioblastoma	49	1.07 ± 0.22
Radiation necrosis	58	0.66 ± 0.14

rADC: relative Apparent Diffusion Coefficient.

## Data Availability

The data presented in this study are available on request from the corresponding author.

## Supplementary Materials

The following supporting information can be downloaded at: https://www.mdpi.com/article/10.3390/cancers16132440/s1, Table S1: Additional descriptive statistics of ADC values.
