# Supplementary material for: The Use of Apparent Diffusion Coefficient Values for Differentiating Bevacizumab-Related Cytotoxicity from Tumor Recurrence and Radiation Necrosis in Glioblastoma
Source: Cancers (Basel). 2024 Jul 2;16(13):2440. doi: 10.3390/cancers16132440 (PMC11240552; doi:10.3390/cancers16132440)
Supplement: Supplementary file 1 [file cancers-16-02440-s001.zip › cancers-3024539-supplementary.pdf]

cancers

MDPI

Supplementary Table S1. Additional descriptive statistics of ADC values.

Group	Descriptive statistics	Lesion	Normal	P value
Bevacizumab	Median (Q_1, Q_3)	270 (201, 280)	668 (629, 700)	<0.001
	Skewness	-0.27	-1.44	0.24
	Kurtosis	0.03	2.52	1.00
Progressive glioblastoma	Median (Q_1, Q_3)	719 (669, 808)	714 (673, 751)	0.08
	Skewness	1.05	-0.31	0.08
	Kurtosis	2.10	1.12	0.64
Radiation necrosis	Median (Q_1, Q_3)	498.5 (424, 542)	720 (693, 770)	<0.001
	Skewness	-0.31	0.32	1.00
	Kurtosis	0.67	0.52	0.83
